# The long-term effectiveness of coronavirus disease 2019 (COVID-19) vaccines: A systematic literature review and meta-analysis

**DOI:** 10.1017/ash.2021.261

**Published:** 2022-02-14

**Authors:** Alexandre R. Marra, Takaaki Kobayashi, Hiroyuki Suzuki, Mohammed Alsuhaibani, Marin L. Schweizer, Daniel J. Diekema, Bruna Marques Tofaneto, Luigi Makowski Bariani, Mariana de Amorim Auler, Jorge L. Salinas, Michael B. Edmond, João Renato Rebello Pinho, Luiz Vicente Rizzo

**Affiliations:** 1 Department of Internal Medicine, University of Iowa Carver College of Medicine, Iowa City, Iowa, United States; 2 Instituto Israelita de Ensino e Pesquisa Albert Einstein, Hospital Israelita Albert Einstein, São Paulo, Brazil; 3 Center for Access & Delivery Research & Evaluation (CADRE), Iowa City Veterans’ Affairs Health Care System, Iowa City, Iowa, United States; 4 Department of Pediatrics, College of Medicine, Qassim University, Qassim, Saudi Arabia; 5 Albert Einstein Medical College, São Paulo, Brazil; 6 Stanford University, Stanford, California, United States; 7 West Virginia University School of Medicine, Morgantown, West Virginia, United States; 8 Research and Development Sector, Clinical Laboratory, Hospital Israelita Albert Einstein, São Paulo, Brazil

## Abstract

**Background:**

Although multiple studies revealed high vaccine effectiveness of coronavirus disease 2019 (COVID-19) vaccines within 3 months after the completion of vaccines, long-term vaccine effectiveness has not been well established, especially after the δ (delta) variant became prominent. We performed a systematic literature review and meta-analysis of long-term vaccine effectiveness.

**Methods:**

We searched PubMed, CINAHL, EMBASE, Cochrane Central Register of Controlled Trials, Scopus, and Web of Science from December 2019 to November 15, 2021, for studies evaluating the long-term vaccine effectiveness against laboratory-confirmed COVID-19 or COVID-19 hospitalization among individuals who received 2 doses of Pfizer/BioNTech, Moderna, or AstraZeneca vaccines, or 1 dose of the Janssen vaccine. Long-term was defined as >5 months after the last dose. We calculated the pooled diagnostic odds ratio (DOR) with 95% confidence interval for COVID-19 between vaccinated and unvaccinated individuals. Vaccine effectiveness was estimated as 100% × (1 − DOR).

**Results:**

In total, 16 studies including 17,939,172 individuals evaluated long-term vaccine effectiveness and were included in the meta-analysis. The pooled DOR for COVID-19 was 0.158 (95% CI: 0.157-0.160) with an estimated vaccine effectiveness of 84.2% (95% CI, 84.0- 84.3%). Estimated vaccine effectiveness against COVID-19 hospitalization was 88.7% (95% CI, 55.8%–97.1%). Vaccine effectiveness against COVID-19 during the δ variant period was 61.2% (95% CI, 59.0%–63.3%).

**Conclusions:**

COVID-19 vaccines are effective in preventing COVID-19 and COVID-19 hospitalization across a long-term period for the circulating variants during the study period. More observational studies are needed to evaluate the vaccine effectiveness of third dose of a COVID-19 vaccine, the vaccine effectiveness of mixing COVID-19 vaccines, COVID-19 breakthrough infection, and vaccine effectiveness against newly emerging variants.

The first coronavirus disease 2019 (COVID-19) vaccine was authorized for emergency use by the US Food and Drug Administration on December 11, 2020.^1^ Over the past several months, research studies have yielded substantial data on short-term (≤ 3 months) vaccine effectiveness^2–4^ against symptomatic COVID-19. For example, the short-term vaccine effectiveness is known to be very high at 95% for the Pfizer/BioNTech COVID-19 vaccine, 94.1% for the Moderna vaccine, 70.4% for the AstraZeneca vaccine, and 66.3% for the Janssen COVID-19 vaccine.^1,5–7^

In the third year of the pandemic, individuals are still at risk of acquiring COVID-19 even with vaccines available.^8,9^ Infection and hospitalization rates among unvaccinated individuals are 5 times and 11–29 times higher than those in vaccinated individuals, respectively.^10,11^ Also, the authorized COVID-19 vaccines protect against the δ variant,^12^ even with increased community transmission.^10,13^

Although these vaccines are effective for a wide range of COVID-19–related outcomes,^1,6,14,15^ the duration of the immune protection following the COVID-19 vaccination is still not well defined,^16–18^ and few studies have assessed the long-term vaccine effectiveness of COVID-19 vaccines.

We reviewed the literature on the long-term vaccine effectiveness of COVID-19 vaccines for COVID-19 and COVID-19 hospitalizations. Pooling the results of published studies allows for more precise estimates of the long-term vaccine effectiveness. The information provided from subset analyses during the δ variant period is significantly important given the ongoing pandemic with this variant.

## Methods

### Systematic literature review and inclusion and exclusion criteria

This review was conducted according to the Preferred Reporting Items for Systematic Reviews and Meta-Analysis (PRISMA) statement^19^ and the Meta-analysis of Observational Studies in Epidemiology (MOOSE) guidelines,^20^ and it was registered on Prospero (https://www.crd.york.ac.uk/PROSPERO/) on September 13, 2021 (registration no. CRD42021278162). The approval of our institutional review board was not required.

The inclusion criteria for studies in this systematic review were as follows: original research manuscripts; published in peer-reviewed scientific journals; involved vaccinated and unvaccinated individuals; evaluated the long-term effectiveness of COVID-19 vaccine; and observational study design. Long-term was defined as >5 months after the second dose for mRNA (Pfizer/BioNTech or Moderna) or AstraZeneca COVID-19 vaccine, or 1 dose of Janssen COVID-19 vaccine. The literature search was limited to December 2019 to November 15, 2021. Editorials, commentaries, and published studies from non–peer-reviewed sources (eg, MedRxiv) were excluded. Studies without comparison between vaccinated and unvaccinated individuals (or other vaccinated control group), and studies without vaccine effectiveness data were also excluded.

### Search strategy

**Fig. 1.**
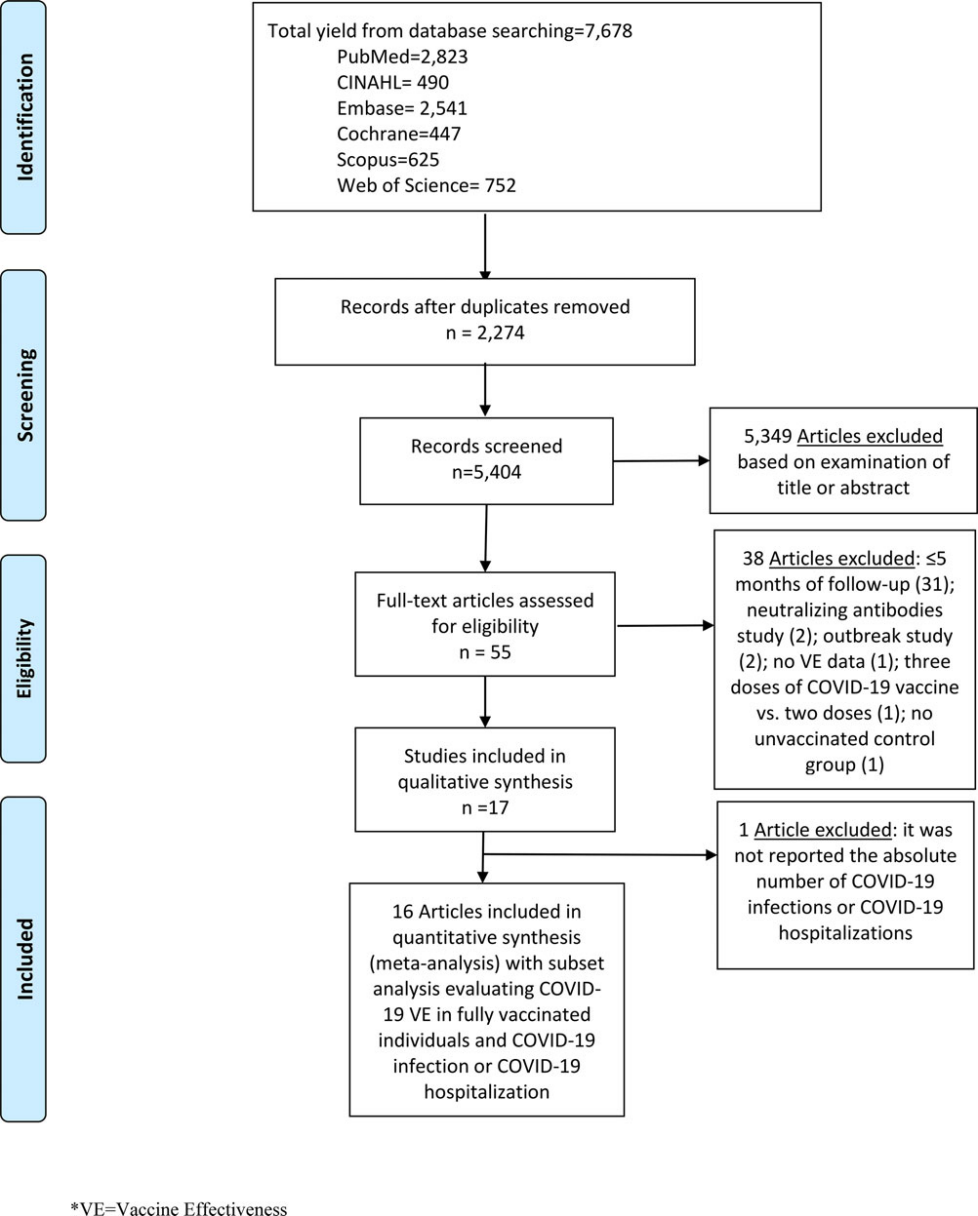
Literature search for articles on the long-term COVID-19 vaccine effectiveness among general population.

We performed literature searches in PubMed, Cumulative Index to Nursing and Allied Health (CINAHL), Embase (Elsevier Platform), Cochrane Central Register of Controlled Trials, Scopus (which includes EMBASE abstracts), and Web of Science. The entire search strategy is described in Supplementary Appendix 1. We reviewed the reference lists of retrieved articles to identify studies that were not identified from the preliminary literature searches. After applying exclusion criteria, we reviewed 55 papers, 17 of which met the inclusion criteria and were included in the systematic literature review (Fig. 1).

### Data abstraction and quality assessment

Titles and abstracts of all articles were screened to assess whether they met inclusion criteria. The reviewers (A.R.M., B.M.T., H.S., L.M.B., M.A., M.A.A., and T.K.) abstracted data for each article. Reviewers resolved disagreements by consensus.

The reviewers abstracted data on study design, population and location, study period (months) and the calendar time, demographic and characteristics of participants, types of COVID-19 vaccine, and the date of whole-genome sequencing if available. Laboratory-confirmed COVID-19 was considered the primary outcome to calculate vaccine effectiveness after 2 doses of a COVID-19 vaccine. COVID-19 hospitalization was considered as a secondary outcome. We collected the hazard ratio (HR), the relative risk (RR), the odds ratio (OR), and vaccine effectiveness with 95% confidence intervals (CIs). We have also described the statistical analysis performed per each study to describe the estimated COVID-19 vaccine effectiveness. Risk of bias was assessed using the Downs and Black scale.^21^ Reviewers followed all questions from this scale as written except for question 27 (a single item on the power subscale scored 0–5), which was changed to a yes or no. Two authors performed component quality analyses independently, reviewed all inconsistent assessments, and resolved disagreements by consensus.^22^

### Statistical analysis

To meta-analyze the extracted data, we calculated the pooled diagnostic odds ratio (DOR) for COVID-19 or COVID-19 hospitalization between vaccinated and unvaccinated individuals. Vaccine effectiveness was estimated as 100% × (1 − DOR). We performed stratified analyses by vaccine type (eg, PfizerBioNTech COVID-19 vaccine [2 doses], Janssen COVID-19 vaccine [1 dose]), by COVID-19 status (ie, COVID-19 or COVID-19 hospitalization), and by the δ variant period.^23–33^ We performed statistical analysis using R version 4.1.0 with *mada* package version 0.5.4.^34^ Analogous to the meta-analysis of the odds ratio methods for the DOR, an estimator of random-effects model following the approach of DerSimonian and Laird is provided by the *mada* package.^34^ For our meta-analysis of estimates of COVID-19 vaccine effectiveness, we used a bivariate random effects model, adopting a similar concept of performing the diagnostic accuracy, which enables simultaneous pooling of sensitivity and specificity with mixed-effect linear modeling while allowing for the trade-off between them.^35,36^ Heterogeneity between studies was evaluated with I^2^ estimation and the Cochran Q statistic test.

## Results

### Characteristics of included studies

In total, 17 studies met the inclusion criteria^23–33,37–42^ and were included in the final review (Table 1). Almost all of these studies were nonrandomized (16 studies), and of these, 12 were retrospective cohort studies.^23,24,26–28,30–33,37,39,40^ Also, 1 study was a prospective cohort study^29^ and 3 studies were case–control studies.^25,38,42^ Only 1 study was a randomized clinical trial.^41^ All but 1 of these studies evaluated the Pfizer/BioNTech vaccine (16 studies).^23–26,28–33,37,39–42^ Of these studies, 13 analyzed the Moderna vaccine^23,24,26,28–32,37,39,40,42^; 7 studies analyzed the Janssen vaccine,^26,27,29,37–39,42^ 1 of which evaluated only the Janssen vaccine^27^; and 3 studies analyzed the AstraZeneca vaccine,^31,37,38^ 1 of which evaluated mixing COVID-19 vaccines.^31^

**Table 1.** Summary of Characteristics of Studies Included in the Systematic Literature Review

First Author, Year, Location	COVID-19 Vaccine	Study Design	Study Period [Dates]	No. of Participants and Characteristics	COVID-19		COVID-19 Hospitalization		Incidence Rate Ratio [IRR], Hazard Ratio [HR], Relative Risk [RR’], or Odds Ratio [OR] (95% CI), and Vaccine Effectiveness (VE) (95% CI)	Statistical Analysis Performed	Downs and Black Score (max= 28)
					Vaccinated 2nd Dose	Control Group [Unvaccinated or Mixing Vaccine]	Vaccinated 2nd Dose	Control Group [Unvaccinated or Mixing Vaccine]			
Bajema 2021, USA	Pfizer/BioNTech, Moderna	Retrospective cohort	6 mo [Feb 2021–Aug 2021]	1,175 participants (432 vaccinated with 2 doses [285 Pfizer/BioNTech; 147 Moderna] vs 743 unvaccinated)	NR	NR	43 (Pfizer/BioNTech); 11 (Moderna)	334	Stratified period of analysis: COVID-19 hospitalization VE=86.8% (80.4%–91.1%) in total 6 months; COVID-19 hospitalization VE=84.1% (74.1%–90.2%) in pre-δ period; COVID-19 hospitalization VE=89.3% (80.1%–94.3%) in during the δ period; Stratified by COVID-19 vaccine type: COVID-19 hospitalization VE for Pfizer/BioNTech = 83.4% (74.0%–89.4%); VE for Moderna = 91.6% (83.5%–95.7%)	Adjusted logistic regression model	21
Bozio 2021, USA	Pfizer/BioNTech and Moderna	Retrospective cohort	9 mo [Jan 20, 2021–Sept 2021]	7,348 participants (6,328 vaccinated with 2 doses [3,736 Pfizer/BioNTech; 2,592 Moderna] vs 1,020 unvaccinated previously infected with COVID-19)	NR	NR	324	89	The odds of laboratory confirmed COVID-19 were higher among previously infected, unvaccinated patients than among vaccinated patients with 2 COVID-19 vaccine doses: Adjusted OR=5.49 (95% CI=2.75–10.99) VE=not reported	Adjusted OR and 95% CIs were calculated using multivariable logistic regression [covariates: age, geographic region, calendar time (days from Jan 1 to hospitalization), and local virus circulation, and weighted based on propensity to be in the vaccinated category]	17
Braeye 2021, Belgium	Pfizer/BioNTech and Moderna, Janssen, and AstraZeneca	Retrospective cohort	5 mo [Jan 21, 2021– Jun 24, 2021]	417,349 participants (8,977 fully vaccinated and 408,372 unvaccinated)	NR	NR	NR	NR	VE for COVID-19: VE=74% (72%–76%) for Pfizer/BioNTech; VE=85% (80%–90%) for Moderna; VE= 61% (29%–84%) for Janssen; VE=53% (12%–84%) for AstraZeneca	VE was defined as the probability of infection given vaccination status (vaccinated and high-risk contacts), compared to an unvaccinated using a Bayesian logistic regression	18
Chemaitelly 2021, Qatar	Pfizer/BioNTech	Case–control	9 mo [Jan 1, 2021–Sept 5, 2021]	990,540 participants (848,240 individuals with PCR-negative SARS-CoV-2 tests were used in the matching with 142,300 individuals with a PCR-confirmed SARS-CoV-2 infection, for whom vaccination records were retrieved)	2,915	112,998	32	4,082	Stratified period of analysis: COVID-19 VE (1st mo after after receipt of 2nd dose, <60 y)=77.8% (76.7%–78.9%); VE (1st mo after after receipt of second dose, >60 y)=71.1% (64.8%–76.3%); VE (≥7 mo after receipt of second dose, <60 y)=24.5% (−0.9% to 43.5%); VE (≥7 mo after receipt of second dose, >60 y)=6.6% (−93.4% to 54.9%) COVID-19 hospitalization VE (1st month after after receipt of second dose, <60 y)=96.9% (94.8%–98.2%); VE (1st month after after receipt of second dose, >60 y)=92.6% (85.5%–96.3%); VE (≥7 mo after receipt of second dose, <60 y)=57.1% (−65.7% to 88.9%); VE (≥7 mo after receipt of second dose, >60 y)=50.0% (−451.4% to 95.5%)	Logistic regression model and the adjusted OR were used to calculate VE as [(1−OR)×100]	21
Cohn 2021, USA	Pfizer/BioNTech, Moderna, and Janssen	Retrospective cohort	8 mo [Feb 2021– Oct 2021]	780,225 participants (462,486 vaccinated with 2 doses [231,724 Pfizer/BioNTech; 230,762 Moderna], and 35,662 with 1 dose of Janssen vs 282,077 unvaccinated)	27,680 (Pfizer/BioNTech); 24,342 (Moderna); 6,945 (Janssen)	72,638	NR	NR	Stratified period of analysis: Mar/2021: COVID-19: VE=86.9% (86.5%–87.3%) for Pfizer/BioNTech; VE=89.2% (88.8%–89.6%) for Moderna; and VE=86.4% (85.2%–87.6%) for Janssen; Sept/2021: COVID-19: VE=43.3% (41.9%–44.6%) for Pfizer/BioNTech; VE=58.0% (56.9%–59.1%) for Moderna; and VE=13.1% (9.2%–16.8%) for Janssen	Cox proportional model	21
Corchado-Garcia 2021, USA	Janssen	Retrospective cohort	5 mo [Feb 27, 2021– Jul 22, 2021]	97,787 participants (8,889 vaccinated patients vs 88,898 unvaccinated patients)	60	2,236	NR	NR	COVID-19: VE=73.6% (65.9%–79.9%) for Janssen	Propensity matched score [covariates: age, sex, zip code, race, ethnicity, and previous number of SARS-CoV-2 PCR tests]. Defined VE as 1−RR of fully vaccinated vs unvaccinated ×100	21
Embi 2021, USA	Pfizer/BioNTech and Moderna	Retrospective cohort	9 mo [Jan 17, 2021– Sept 5, 2021]	89,217 participants (40,020 vaccinated with 2 doses [29,456 immunocompetent, and 10,564 immunocompromised] and 49,197 unvaccinated [39,660 immunocompetent, and 9,537 immunocompromised)	NR	NR	12,498	10,980	VE=88% (86%–89%) for Pfizer/BioNTech: immunocompetent; and VE=71% (65%–76%) for Pfizer/BioNTech:immunocompromised VE=93% (92%–94%) for Moderna: immunocompetent; VE=81% (76%–85%) for Moderna and immunocompromised	Adjusted VE was estimated by using a test-negative design comparing the odds of a positive test result for SARS-CoV-2 between fully vaccinated and unvaccinated patients using multivariable logistic regression models	21
Fowlkes 2021, USA	Pfizer/BioNTech, Moderna, and Janssen	Prospective cohort	8 mo [Dec 2020– Aug 2021]	7,112 participants (2,976 vaccinated with 2 doses, and 4,136 unvaccinated)	34	194	NR	NR	Stratified period of analysis: COVID-19 symptomatic infection VE=80% (69%–88%) in total 8 months; COVID-19 symptomatic infection VE=91% (81%–96%) in pre-δ period; COVID-19 symptomatic infection VE=66% (26%–84%) in during the δ period	Cox proportional hazards models	20
Kissling 2021, Europe (England, France, Ireland, Netherlands, Portugal, Scotland, Spain, and Sweden)	Pfizer/BioNTech and Moderna, Janssen, and AstraZeneca	Case–control	6 mo [Dec 2020– May 2021]	4,964 participants aged ≥65 y (592 cases, and 4,372 controls with 693 fully vaccinated, and 2, 866 unvaccinated)	14	508	NR	NR	VE=89.0% (79.0%–94.0%) for all COVID-19 vaccines	Logistic regression model and the adjusted OR were used to calculate VE as [(1−OR)×100]	21
Nanduri 2021, USA	Pfizer/BioNTech and Moderna	Retrospective cohort	5.5 mo [Feb 15, 2021– Aug 1, 2021]	10,428,783 aggregate weekly resident counts (7,807,798 vaccinated with 2 doses [5,174,098 Pfizer/BioNTech; 2,633,700 Moderna], and 1,089,539 with other vaccination status vs 1,531,446 unvaccinated)	3,905 (for mRNA COVID-19 vaccine)	2,113	NR	NR	Stratified period of analysis (mRNA vaccine): Pre-δ period: VE=74.7% (70.0%–78.8%) Intermediate period: VE=67.5% (60.1%–73.5%) δ period: VE=53.1% (49.1%–56.7%)	Defined VE as 1-RR (rate ratio) of fully vaccinated (2 doses) vs unvaccinated ×100 derived from a Poisson regression	15
Nordstrom 2021, Sweden	Pfizer/BioNTech (A), Moderna (B) and AstraZeneca (C)	Retrospective cohort	7 mo [Follow-up of 214 days up to Aug 23, 2021]	721,877 participants (541,071 vaccinated with 2 doses) [94,569 A+C vaccination; 16,402 B+C vaccination, and 430,100 C+C vaccination] vs 180,716 unvaccinated)	A+C vaccine: 170; B+C vaccine: 17; C+C vaccine: 446	unvaccinated (vs A+C): 259; unvaccinated (vs B+C): 47; unvaccinated (vs C+C): 323	A+C vaccine: 1; B+C vaccine: 0; C+C vaccine: 2	Unvaccinated individuals: 16	Adjusted VE for COVID-19: VE=67.0% (59.0%–73.0%) for A+C vaccine; VE=79.0% (62.0%–88.0%) for B+C vaccine; VE=50.0% (41.0%–58.0%) for C+C vaccine	Cox proportional hazard model, Adjusted HR were used to calculate VE as [(1−HR) ×100]	22
Nunes 2021, Portugal	Pfizer/BioNTech and Moderna	Retrospective cohort	6 mo [Feb 2020– Aug 2021]	For the mRNA VE analysis: 878,489 participants aged >65–79 years (753,151 vaccinated with 2 doses [641,119 Pfizer/BioNTech; 112,032 Moderna] vs 125,338 unvaccinated); and 460,820 participants aged ≥80 years (433,878 vaccinated with 2 doses [378,312 Pfizer/BioNTech; 55,566 Moderna] vs 26,942 unvaccinated)	NR	NR	mRNA vaccines: aged 65–79 y = 11; ≥80 y= 43	Unvaccinated: aged 65–79 y= 169; Aged ≥80 y = 734	Stratified period of analysis (mRNA COVID-19 vaccine) for COVID-19 hospitalization: Aged 65–79 years: VE=94.0% (88.0%–97.0%); Aged ≥80 years: VE=82.0% (72.0%–89.0%); for COVID-19 death prevention: Aged 65–79 years: VE=96.0% (92.0%–98.0%); Aged ≥80 years: VE=81.0% (74.0%–87.0%)	Multivariable Cox proportional hazard model. Adjusted HR were used to calculate VE as [(1−HR)×100]	21
Self 2021, USA	Pfizer/BioNTech (A), Moderna (B) and Janssen (C)	Retrospective cohort	8.5 mo [Mar 11, 2021– Aug 15, 2021]	3,689 participants (1,214 vaccinated with 2 doses [738 Pfizer/BioNTech; 476 Moderna], and 113 with 1 dose of Janssen vs 2,362 unvaccinated)	NR	NR	A:128 B:54 C:37	1,463	Stratified period of analysis: COVID-19 hospitalization: Pfizer/BioNTech: VE=88.0% (85.0%–91.0%) Moderna: VE=93.0% (91.0%–95.0%) Janssen: VE=71.0% (56%–81%) For ≥120 d COVID-19 hospitalization: Pfizer/BioNTech: VE=77.0% (67.0%–84.0%) Moderna: VE=92.0% (87.0%–96.0%) Janssen: VE=NR	Defined VE as 1−OR of fully vaccinated vs unvaccinated ×100 VE against COVID-19 hospitalization was estimated using logistic regression	21
Tande 2021, USA	Pfizer/BioNTech and Moderna	Retrospective cohort	8.5 mo [Jan 1, 2021– Aug 15, 2021]	P1 (Jan 1–Mar 31): 1,948 fully vaccinated vs 17,764 unvaccinated P2 (Apr 1–May 31): 7,751 fully vaccinated vs 4,750 unvaccinated; P3 (Jun 1–Aug 15): 10,551 fully vaccinated vs 7,057 unvaccinated	P1: 2 (A) P2: 23 (A) P3: 36 (A)	P1: 222 (A) P2: 62 (A) P3: 78 (A)	NR	NR	Adjusted VE for asymptomatic (A) COVID-19: VE=71.0% (61.0%–78.0%); P1:VE=91.0% (72.0%–98.0%); P2:VE=71.0% (53.0%–83.0%); P3:VE=63.0% (44.0%–76.0%)	Defined VE as 1−RR’ (relative risk) of fully vaccinated (2 doses) vs unvaccinated ×100, using mixed effect modeling	21
Tartof 2021, USA	Pfizer/BioNTech	Retrospective cohort	8 mo [Dec 14, 2020–Aug 8, 2021]	3,333,478 participants (1,043,289 fully vaccinated; and 2,290,189 unvaccinated)	3,355	151,855	NR	NR	Adjusted VE for COVID-19: VE=73.0% (72.0%–74.0%); Adjusted VE for COVID-19 hospitalization: VE=90.0% (89.0%–92.0%); Adjusted VE for COVID-19 by the δ variant: VE=75.0% (71.0%–78.0%);	Cox proportional hazard model, Adjusted HR were used to calculate VE as [(1−HR) ×100]	22
Thomas 2021, USA	Pfizer/BioNTech	RCT	6 mo [6 mo after the initiation of the vaccination in Jul 27, 2000]	42,094 participants (20,998 fully vaccinated; and 21,096 placebo)	77	850	NR	NR	Stratified period of analysis: COVID-19 VE=91.3% (89.0%–93.2%); VE ≥4 mo after receipt of 2nd dose=83.7% (74.7%–89.9%)	Defined VE as 1−IRR (incidence rate ratio) of fully vaccinated (2 doses) vs placebo group ×100, using a Bayesian logistic regression	27
Thompson 2021, USA	Pfizer/BioNTech (A), Moderna (B) and Janssen(C)	Case–control	6.5 mo [Jan 1, 2020–Jun 22, 2021]	41,552 hospitalizations of inpatients aged ≥50 years (11,292 vaccinated with Pfizer/BioNTech [8,500 vaccinated with 2 doses]; 9,147 vaccinated with Moderna [6,374 vaccinated with 2 doses], and 707 with 1 dose of Janssen vs 20,406 unvaccinated)	NR	NR	A:163 B:95 C:30	3,695 (unvaccinated for A and B); 2,006 (unvaccinated for C)	Stratified period of analysis: COVID-19 hospitalization: Pfizer/BioNTech: VE=87.0% (85.0%–90.0%) Moderna: VE=91.0% (89.0%–93.0%) Janssen VE=68.0% (50%–79%)	Logistic regression model and the use of a test negative design to calculate VE as [(1−OR)×100]	21

Note. A, asymptomatic; S, symptomatic; SD, standard deviation; IQR, interquartile range; IRR, incidence rate ratio; HR, hazard ratio [HR]; RR’, relative risk; OR, odds ratio; 95% CI, 95% confidence interval; VE, vaccine effectiveness; NR, not reported; N, number reported; RCT, randomized controlled trial.

**Table 2.** Subset Analyses Evaluating Long-Term COVID-19 Vaccine Effectiveness Among Fully Vaccinated Individuals

Vaccine	Outcome	No. of Studies Included	Participants, No.	Pooled Diagnostic Odds Ratio [DOR] (95% CI)	I^2^ test for heterogeneity, %	Vaccine Effectiveness, % (95% CI)^a^
mRNA or viral vector	Infection	10	16,456,882	0.158 (0.157–0.160)	0	84.2 (84.0–84.3)
Pfizer/BioNTech	Infection	5	15,575,120	0.185 (0.184–0.187)	0	81.5 (81.3–81.6)
mRNA or viral vector	Infection during the δ variant period	4	11,476,256	0.388 (0.367–0.410)	0	61.2 (59.0–63.3)
mRNA or other vaccines	Hospitalization	8	3,194,708	0.113 (0.029–0.442)	0	88.7 (55.8–97.1)
Pfizer/BioNTech	Hospitalization	6	1,133,521	0.146 (0.140–0.152)	51	85.4 (84.8–86.0)
Moderna	Hospitalization	5	142,981	0.102 (0.096–0.108)	32	89.8 (89.2–90.4)

Note. CI, confidence interval; mRNA, Pfizer/BioNTech and Moderna; viral vector, AstraZeneca, and Janssen.

a Vaccine effectiveness was estimated as 100% × (1 − DOR).

Fully vaccinated is defined as receiving 2 doses of Pfizer/BioNTech, Moderna, or AstraZeneca vaccine, or 1 dose of Janssen vaccine.

Most of the studies included in our review were conducted in the United States (12 studies)^23,24,26–30,33,39–42^; 1 study was a multicenter study performed in Europe (assembling data from England, France, Ireland, Netherlands, Portugal, Scotland, Spain, and Sweden)^38^; and 1 study was performed in each of these countries: Belgium,^37^ Qatar,^25^ Sweden,^31^ and Portugal.^32^ All studies were performed between December 2020 and October 2021.^23–33,37–42^

Moreover, 10 studies evaluated long-term vaccine effectiveness for COVID-19,^25–27,29–31,33,38,40,41^ 8 studies evaluated long-term vaccine effectiveness for COVID-19 hospitalizations,^23–25,28,31,32,39,42^ with 2 studies overlapping.^25,31^ The study duration varied from 5 to 14 months.^23–33,37–42^

Furthermore, 13 studies reported genomic surveillance data.^23–26,28–33,37,38^ Also, 11 studies reported detecting the new SARS-CoV-2 B.1.617.2 δ (delta) variant^23–33^; 7 studies reported only δ variant during the long-term vaccine effectiveness evaluation^23,24,26,28–31^; 2 studies reported the B.1.1.7 α (alpha) variant and δ variant^27,32^; 1 study reported the B.1.351 β (beta) variant and δ variant,^25^ and 1 study reported α, β, γ (gamma or P.1), and δ variants.^25^

Studies varied with regards to the type of statistical analysis performed. Nine studies used logistic regression^23–25,28,37–39,41,42^; 5 studies used Cox proportional hazard analysis^26,29,31–33^; 1 study used propensity matched scoring^27^; 1 study used Poisson distribution for adjusted logistic regression^30^; and 1 study used mixed-effects modeling.^40^

Regarding the quality assessment scores of the 17 included studies, >75% of the studies (13 studies) were considered good quality (ie, 19–23 of 28 possible points) per the Downs and Black quality tool.^23,25–29,31–33,38–40,42^ Also, 3 studies were considered fair quality (ie, 14–18 points)^24,30,37^ and 1 study was considered high quality (ie, >24 points).^41^

### Results pooled by COVID-19 vaccine type and COVID-19 outcome

Overall, we included 17,939,172 individuals from 16 studies in the meta-analysis.^23–33,38–42^ Among them, 10 studies evaluated the long-term vaccine effectiveness of mRNA or viral vector vaccines (ie, AstraZeneca or Janssen).^25–27,29–31,33,38,40,41^ The estimated long-term vaccine effectiveness for COVID-19 was 84.2% (95% CI, 84.0%–84.3%). Also, 5 studies evaluated the long-term vaccine effectiveness of the Pfizer/BioNTech vaccine,^25,26,30,33,41^ and 2 studies evaluated the Moderna vaccine.^26,30^ The estimated long-term vaccine effectiveness against COVID-19 of the Pfizer/BioNTech COVID-19 vaccine was 81.5% (95% CI, 81.3%–81.6%). Furthermore, 4 studies evaluated vaccine effectiveness of the mRNA or viral vector vaccines during the δ variant period^25,29,30,40^; 2 studies evaluated vaccine effectiveness of the Pfizer/BioNTech COVID-19 vaccine only^25,30^, and 2 studies reported vaccine effectiveness of the Moderna COVID-19 vaccine only.^25,30^ The estimated long-term vaccine effectiveness for COVID-19 with mRNA or viral vector vaccines during the δ variant–dominant period was 61.2% (95% CI, 59.0%–63.3%).

Among the 16 studies, 8 studies evaluated the long-term vaccine effectiveness of mRNA or viral vector vaccines for COVID-19 hospitalization.^23–25,28,31,32,39,42^ The estimated long-term vaccine effectiveness against COVID-19 was 88.7% (95% CI, 55.8%–97.1%). In stratified analyses, 6 studies evaluated long-term vaccine effectiveness for COVID-19 hospitalization with the Pfizer/BioNTech vaccine,^23–25,28,39,42^ and 5 studies with the Moderna vaccine.^23,24,28,39,42^ The estimated long-term vaccine effectiveness for COVID-19 hospitalization with the Pfizer/BioNTech vaccine was 85.4% (95% CI, 84.8%–86.0%). The estimated long-term vaccine effectiveness for COVID-19 hospitalization with the Moderna vaccine was 89.8% (95% CI, 89.2%–90.4%). Only 1 study evaluated COVID-19 hospitalization during the δ variant period with mRNA vaccines.^24^ This study did not report the COVID-19 vaccine effectiveness but reported that the adjusted odds of COVID-19 was higher among unvaccinated and previously infected patients compared with fully vaccinated individuals (adjusted odds ratio, 5.49; 95% CI, 2.75–10.99).^24^

The results of meta-analyses were homogeneous for COVID-19 with mRNA or viral vector vaccines (heterogeneity *P* = .76; I^2^ = 0%); studies evaluating individuals vaccinated with the Pfizer/BioNTech vaccine alone (heterogeneity *P* = .55; I^2^ = 0%); and studies evaluating individuals vaccinated with mRNA or viral vector vaccines during the δ variant period (heterogeneity *P* = .50; I^2^ = 0%).

Meta-analysis results were also homogeneous for COVID-19 hospitalization (studies evaluating individuals vaccinated with mRNA or viral vector vaccines (heterogeneity *P* = .67; I^2^ = 0%); and studies evaluating individuals vaccinated with the Moderna vaccine alone (heterogeneity *P* = .28; I^2^ = 20%). However, results were not homogenous for studies of COVID-19 hospitalization only evaluating individuals vaccinated with the Pfizer/BioNTech vaccine alone (heterogeneity *P* = .07; I^2^ = 51%) or for studies of COVID-19 hospitalization only evaluating individuals vaccinated with the Moderna vaccine alone (heterogeneity *P* = .21; I^2^ = 32%).

## Discussion

This systematic literature review and meta-analysis showed that the long-term of vaccine effectiveness with COVID-19 vaccines (primarily the mRNA vaccines) for COVID-19 and COVID-19 hospitalization were high at 84.2% and 88.7%, respectively. However, the long-term vaccine effectiveness against COVID-19 during the δ-variant–dominant period was lower at 61.2%. These results suggest that 2 doses of the COVID-19 vaccine may lose effectiveness after a few months, and more prospective studies are needed to investigate the short- and long-term vaccine effectiveness after the third dose of the COVID-19 vaccines.

A growing body of early global research shows that the authorized COVID-19 vaccines remain highly protective against the disease’s worst outcomes over time with some exceptions among older and immunocompromised people.^43,44^ In our systematic literature review, we analyzed only the estimated pooled vaccine effectiveness for the mRNA COVID-19 vaccines and the viral vector COVID-19 vaccines. These are the first COVID-19 vaccines authorized by the FDA and around the world,^45–48^ and they prevent COVID-19 and COVID-19 hospitalization.^2,4,10,12,15,49^ The long duration of the studies (from 5 to 14 months, as shown in Table 1) included in our systematic literature review helps to better elucidate the long-term vaccine effectiveness in the context of a global pandemic with new SARS-CoV-2 variants^12,13^ and to better understand that the decrease of vaccine effectiveness is associated with a waning of humoral immune response after a few months.^13,17^ Although the overall long-term vaccine effectiveness against COVID-19 and COVID-19 hospitalization were moderately high (∼80%), a number of published studies demonstrated significantly lower vaccine effectiveness (∼60%) during the δ-variant period.^25,26,29,30,39,41^

Our systematic review included 11 studies evaluating the widespread circulation of the δ variant contributing to the majority of recent COVID-19 and COVID-19 hospitalizations.^23–33^ The studies in this systematic review antedate the emergence of the B.1.1.529 (omicron) variant announced by the World Health Organization (WHO) on November 26, 2021.^50^ We need more studies on the SARS-CoV-2 variants of concerns (VOC) that have multiple spike-protein changes and that may be more infectious or cause more severe disease than other circulating variants.^51^ Some deletions in the spike-protein gene can alter the shape of the spike and may help it evade antibodies.^52^ No COVID-19 vaccine is 100% effective against SARS-CoV-2 infection, as demonstrated by breakthrough infections,^8,53^ but they are highly effective at preventing severe disease and death.^25^ Although the long-term vaccine effectiveness was not as high as the short-term vaccine effectiveness, it is not clear whether the waning of immunity is due to the passage of time or the coincident spread of the δ variant (from June to September 2021).^23–33^

Our study had several limitations. Most of the studies included in the meta-analysis were observational studies, which are subject to multiple biases.^54^ However, this is the most common study design in the infection prevention literature.^54^ None of the included studies reported possible adverse events after vaccine administration. We could not perform further analyses stratified by immunocompromised status due to the limited number of studies. Only 1 study compared immunocompromised individuals to immunocompetent individuals and reported that the effectiveness of mRNA vaccination against COVID-19 hospitalization was lower (77%) among immunocompromised individuals than among immunocompetent individuals (90%).^28^ Because our study focused on the long-term vaccine effectiveness after the second dose, we could not evaluate the impact of a third dose. Because of the low number of included studies of viral vector vaccines, it was not possible to perform a stratified analysis for these. It was not possible to evaluate the long-term vaccine effectiveness of the Moderna vaccine against COVID-19 because there were not enough studies.^26,30^ There are not enough studies comparing each 1 of the 2 mRNA vaccines to draw conclusions about the vaccine effectiveness for COVID-19 during the δ variant dominant period.^25,30^ Also, it was not possible to evaluate the COVID-19 hospitalization vaccine effectiveness during the δ-variant–dominant period. It was not possible to make any conclusions about the long-term vaccine effectiveness of mixing vaccines because just 1 study assessed this.^31^ From that study, mixing COVID-19 vaccines (first dose with the AstraZeneca vaccine adding a mRNA prime-boost showed a higher vaccine effectiveness (68%) than that of 2 doses of AstraZeneca vaccine (50%).^31^ Lastly, each study used a different approach to report the incidence of COVID-19 (eg, incidence rate per person years). Therefore, we decided to perform our meta-analysis and stratified analysis with a bivariate approach to preserve the 2-dimensional nature of the original data from the selected studies.^23–33,38–42^

In conclusion, COVID-19 vaccines can effectively prevent COVID-19 and COVID-19 hospitalization for a relatively long period. These vaccines are also effective in preventing COVID-19 during the δ-variant period, though vaccines were less effective. These data are very important to help motivate individuals to seek vaccination. More observational studies are needed to evaluate other types of COVID-19 vaccine (eg, viral vector or inactivated virus) effectiveness, vaccine effectiveness of a third dose, vaccine effectiveness of mixing COVID-19 vaccines, COVID-19 breakthrough infection after vaccination, and genomic surveillance for better understanding vaccine effectiveness against the new viral variants.

## Supplementary material

For supplementary material accompanying this paper visit https://doi.org/10.1017/ash.2021.261. click here to view supplementary material
