# Supplementary material for: The long-term effectiveness of coronavirus disease 2019 (COVID-19) vaccines: A systematic literature review and meta-analysis
Source: Antimicrob Steward Healthc Epidemiol. 2022 Feb 14;2(1):e22. doi: 10.1017/ash.2021.261 (PMC9614898; doi:10.1017/ash.2021.261)
Supplement: Supplementary file 1 [file S2732494X21002618sup001.docx]

| **Supplementary Appendix 1.** Search terms and strategies | | | |
| --- | --- | --- | --- |
| **Vaccines** | **COVID 19** | **Efficacy** | **General population** |
| Medical subject headings (PubMed)  Covid-19 Vaccines  RNA, Messenger  Vaccines  Vaccination  Immunization | Medical subject headings (PubMed)  COVID-19  SARS-CoV-2  Coronavirus Infections (no explode) | Medical subject headings  (PubMed)  Treatment outcome | No good terms to capture, but will try to exclude via NOT string:  Drug related side effects and adverse reactions  Adverse effects subheading  Case reports  Surveys and questionnaires  Attitude to health  Monoclonal antibodies |
| Emtree (Embase)  SARS CoV-2 Vaccine  Messenger RNA  SARS-CoV-2 antibody  Vaccine  Vaccination  Immunization | Emtree (Embase)  Coronavirus disease 2019  Coronavirus infection (no explode) | Emtree  Treatment outcome  Drug efficacy | Emtree  Monclonal antibody  Attitude to health  Questionnaire  Case report  Adverse drug reaction  Adverse drug reaction subheading  Animal cell  Animal model  Animal tissue  Nonhuman  Animal experiment |
| CINAHL headings  COVID 19 vaccines  RNA, Messenger  Vaccines  Immunization (no explode, includes anti vacc movement and vacc rates) | CINAHL headings  COVID-19  SARS-CoV-2  Coronavirus infections (no explode) | CINAHL headings  Treatment outcomes | CINAHL Headings |
| Free text words/phrases  Vaccine(s)  Vaccination(s)  Pfizer  Bnt162b2  Moderna  immunization(s)  variolation(s)  Immunologic stimulation  Immunostimulation  mRNA-1273  ChAdOx1-S  AZD1222  AstraZeneca/Oxford  Janssen  Johnson&Johnson  Ad26COVS1  JNJ-78436735  Gamaleya  Sputnik V  Sinovac  Corona Vac | Free text words/phrases  Covid  Covid 19  Corona virus  Coronavirus  2019-nCoV  SARS-CoV-2  2019-nCoV | Free text words/phrases  Effectiveness  Efficacy  Effective  Antibody response  Antibody levels  IgG  Neutralizing antibodies  Success | Free text words/phrases  Hesitancy  Attitudes  Beliefs  Perceptions  Reluctance |

**PubMed initial attempt 10/12/2021**

((("Treatment Outcome"[Mesh]) OR (efficacy[Title] OR effectiveness[Title] OR effective[Title] OR IgG[Title/Abstract] OR antibody response[Title/Abstract] OR antibody level[Title/Abstract] OR antibody levels[Title/Abstract] OR neutralizing antibodies[Title/Abstract] OR success[Title/Abstract])) AND ((((vaccine[Text Word] OR vaccines[Text Word] OR vaccination[Text Word] OR vaccinations[Text Word] OR immunization[Text Word] OR immunizations[Text Word] OR variolation[Text Word] OR variolations[Text Word] OR immunologic stimulation[Text Word] OR immunostimulation[Text Word] OR pfizer[Text Word] OR bnt162b2 [Text Word] OR moderna[Text Word]) AND (Covid[Text Word] OR Covid 19[Text Word] OR coronavirus[Text Word] OR corona virus[Text Word] OR 2019 ncov[Text Word] OR sars cov 2[Text Word] OR 2019 nCov[Text Word]))) OR (("RNA, Messenger"[Mesh] OR "Vaccines"[Mesh] OR "Vaccination"[Mesh] OR "Immunization"[Mesh]) AND ("COVID-19"[Mesh] OR "SARS-CoV-2"[Mesh] OR "Coronavirus Infections"[Mesh:NoExp]))) OR ("COVID-19 Vaccines"[Mesh]))) NOT (((("Drug-Related Side Effects and Adverse Reactions"[Mesh]) OR "adverse effects" [Subheading]) OR "Case Reports" [Publication Type]) OR "Surveys and Questionnaires"[Mesh])

= 6790

**PubMed Revised 11/12/21**

#1

("RNA, Messenger"[Mesh] OR "Vaccines"[Mesh] OR "Vaccination"[Mesh] OR "Immunization"[Mesh]) AND ("COVID-19"[Mesh] OR "SARS-CoV-2"[Mesh] OR "Coronavirus Infections"[Mesh:NoExp])

#2

 (vaccine[Text Word] OR vaccines[Text Word] OR vaccination[Text Word] OR vaccinations[Text Word] OR immunization[Text Word] OR immunizations[Text Word] OR variolation[Text Word] OR variolations[Text Word] OR immunologic stimulation[Text Word] OR immunostimulation[Text Word] OR pfizer[Text Word] OR bnt162b2 [Text Word] OR moderna[Text Word]) AND (Covid[Text Word] OR Covid 19[Text Word] OR coronavirus[Text Word] OR corona virus[Text Word] OR 2019 ncov[Text Word] OR sars cov 2[Text Word] OR 2019 nCov[Text Word])

#3

“COVID-19 Vaccines"[Mesh]

#4

"Treatment Outcome"[Mesh] OR efficacy[Title] OR effectiveness[Title] OR effective[Title] OR IgG[Title/Abstract] OR antibody response[Title/Abstract] OR antibody level[Title/Abstract] OR antibody levels[Title/Abstract] OR neutralizing antibodies[Title/Abstract] OR success[Title/Abstract] OR protection [Title] OR protect [Title] OR protecting [Title] OR prevention [Title] OR prevent [Title]

#5

"Drug-Related Side Effects and Adverse Reactions"[Mesh] OR "adverse effects" [Subheading] OR "Case Reports" [Publication Type] OR "Surveys and Questionnaires"[Mesh] OR "Antibodies, Monoclonal"[Mesh] OR "Attitude to Health"[Mesh] OR preprint[pt] OR hesitance [Title] OR hesitancy [Title] OR hesitation [Title] OR attitude [Title] OR attitudes [Title] OR acceptance [Title] OR compliance[Title] OR accept  [Title] OR acceptability [Title] OR perception [Title] OR perceptions[Title]  OR influence [Title] OR influencing [Title] OR intent[Title]  OR intention[Title]  OR intentions [Title] OR perspective [Title] OR perspectives[Title]  OR opinion [Title] OR opinions [Title] OR willingness [Title] OR receptivity[Title]  OR barriers[Title]  OR barrier[Title]  OR motivation[Title]  OR behavior[Title] OR behaviors [Title] OR belief[Title] OR beliefs[Title] OR decision[Title] OR decisions[Title] OR pregnant[Title] OR pregnancy[Title] OR cost[Title] OR economic[Title] OR development[Title] OR supply[Title] OR distribution[Title] OR allocation[Title] OR prioritization[Title] OR mandate[Title] OR mandatory[Title] OR policy[Title] OR policies[Title] OR canine[Title] OR canines [Title] OR dog[Title] OR dogs[Title] OR veterinary[Title] OR mice[Title] OR mouse[Title] OR rodent[Title] OR rodents[Title] OR nonhuman[Title] OR porcine[Title] OR swine[Title] OR primate[Title] OR primates[Title] OR macaques[Title] OR rat[Title] OR rats[Title] OR animal model[Title] OR animal models[Title]

#6

"Animals"[MeSH Terms] NOT ("Animals"[MeSH Terms] AND "Humans"[MeSH Terms])

((#1 OR #2 OR #3) AND #4) NOT (#5 OR #6)

=2823 (513 reviews)

**Embase 11/12/21**

#1

'sars-cov-2 vaccine'/exp

#2

('messenger rna'/exp OR 'sars-cov-2 antibody'/exp OR 'vaccine'/exp OR 'vaccination'/exp OR 'immunization'/exp OR vaccine*:ab,ti OR vaccination*:ab,ti OR pfizer:ab,ti OR moderna:ab,ti OR immunization*:ab,ti OR variolation*:ab,ti OR 'immunologic stimulation':ab,ti OR immunostimulation:ab,ti OR gamaleya:ab,ti OR 'sputnik v':ab,ti OR sinovac:ab,ti OR 'corona vac':ab,ti OR astrazeneca:ab,ti OR 'azd1222':ab,ti OR 'mrna-1273':ab,ti OR janssen:ab,ti OR 'johnson & johnson':ab,ti OR 'jnj-78436735':ab,ti) AND ('coronavirus disease 2019'/exp OR 'coronavirus infection'/de OR covid:ab,ti OR 'covid 19':ab,ti OR 'corona virus':ab,ti OR coronavirus:ab,ti OR 'sars-cov-2':ab,ti OR '2019-ncov':ab,ti)

#3

'drug efficacy'/exp OR 'treatment outcome'/exp OR effectiveness:ti OR efficacy:ti OR effective,ti OR 'antibody response':ab,ti OR 'antibody levels':ab,ti OR igg:ab,ti OR 'neutralizing antibodies':ab,ti OR success:ti OR prevent:ti OR prevention:ti OR protect:ti OR protection:ti OR protecting:ti

#4

'monoclonal antibody'/exp OR 'attitude to health'/exp OR 'questionnaire'/exp OR 'case report'/exp OR 'adverse drug reaction'/exp OR 'adverse drug reaction'/lnk OR 'animal cell'/de OR 'animal model'/de OR 'animal tissue'/de OR 'nonhuman'/de OR 'animal experiment'/exp OR (hesitance OR hesitancy OR hesitation  OR attitude OR attitudes OR acceptance OR compliance OR accept  OR acceptability  OR perception OR perceptions  OR influence  OR influencing  OR intent  OR intention OR intentions  OR perspective  OR perspectives OR opinion  OR opinions  OR willingness  OR receptivity  OR barriers  OR barrier  OR motivation  OR behavior OR behaviors OR belief OR beliefs OR decision OR decisions OR pregnant OR pregnancy OR cost OR economic OR development OR supply OR distribution OR allocation OR prioritization OR mandate OR mandatory OR policy OR policies OR canine OR canines OR dog OR dogs OR veterinary OR mice OR mouse OR rodent OR rodents OR nonhuman OR porcine OR swine OR primate OR primates OR macaques OR rat OR rats OR ‘animal model’ OR ‘animal models’):ti

(#1 OR #2) AND #3) NOT #4=2541 (285 reviews)

**CINAHL 11/11/21**

#1

MH "COVID-19 Vaccines"

#2

(MH "RNA, Messenger" OR MH "Vaccines+" OR MH "Immunization" OR Vaccine* OR Vaccination* OR  Pfizer OR  Moderna OR immunization* OR variolation* OR “immunologic stimulation" OR  Immunostimulation OR Gamaleya OR "Sputnik V" OR  Sinovac OR "Corona Vac" OR AstraZeneca OR Janssen OR "AZD1222" OR "mRNA-1273" OR Janssen OR "Johnson & Johnson" OR "JNJ-78436735") AND (MH "Coronavirus Infections" OR MH "COVID-19" OR MH "SARS-CoV-2" OR Covid OR "Covid 19" OR "Corona virus" OR Coronavirus OR "2019-nCoV" OR "SARS-CoV-2" OR  "2019-nCoV")

#3

MH "Treatment Outcomes+" OR TI (Effective* OR efficacy OR “antibody response” OR antibody levels” OR IgG OR “neutralizing antibodies” OR success OR prevent* OR protect*) OR AB (Effective* OR efficacy OR “antibody response” OR antibody levels” OR IgG OR “neutralizing antibodies” OR success OR prevent* OR protect*)

#4

TI (hesitance OR hesitancy OR hesitation  OR attitude OR attitudes OR acceptance OR compliance OR accept  OR acceptability  OR perception OR perceptions  OR influence  OR influencing  OR intent  OR intention OR intentions  OR perspective  OR perspectives OR opinion  OR opinions  OR willingness  OR receptivity  OR barriers  OR barrier  OR motivation  OR behavior OR behaviors OR belief OR beliefs OR decision OR decisions OR pregnant OR pregnancy OR cost OR economic OR development OR supply OR distribution OR allocation OR prioritization OR mandate OR mandatory OR policy OR policies) OR MH "Attitude to Health+" OR MH "Surveys+" OR MH "Questionnaires+" OR MH "Survey Research" OR MH "Case Studies" OR MH "Adverse Drug Event+" OR MH "Antibodies, Monoclonal+")

#1 OR #2 AND #3 NOT #4= 490 (41 reviews)

**Scopus  11/12/21**

TITLE(Vaccine* OR Vaccination* OR Pfizer OR Moderna OR immunization* OR variolation* OR "immunologic stimulation" OR Immunostimulation OR Gamaleya OR "Sputnik V" OR Sinovac OR "Corona Vac" OR AstraZeneca OR Janssen OR "AZD1222" OR "mRNA-1273" OR Janssen OR "Johnson & Johnson" OR "JNJ-78436735") AND TITLE-ABS(covid OR "Covid 19" OR "Corona virus" OR Coronavirus OR 2019nCoV OR SARSCoV2 OR 2019-nCoV) AND TITLE(Effective* OR efficacy OR "antibody response" OR "antibody levels" OR IgG OR "neutralizing antibodies" OR success OR protect* OR prevent*) AND NOT TITLE(hesitance OR hesitancy OR hesitation OR attitude OR attitudes OR acceptance OR compliance OR accept OR acceptability OR perception OR perceptions OR influence OR influencing OR intent OR intention OR intentions OR perspective OR perspectives OR opinion OR opinions OR willingness OR receptivity OR barriers OR barrier OR motivation OR behavior* OR belief* OR questionnaire* OR survey* OR "case report" OR adverse OR "monoclonal antibodies" OR attitudes OR acceptance OR compliance OR accept OR acceptability OR perception OR perceptions OR influence OR influencing OR intent OR intention OR intentions OR perspective OR perspectives OR opinion OR opinions OR willingness OR receptivity OR barriers OR barrier OR motivation OR behavior OR behaviors OR belief OR beliefs OR decision OR decisions OR pregnant OR pregnancy OR cost OR economic OR development OR supply OR distribution OR allocation OR prioritization OR mandate OR mandatory OR policy OR policies) AND ( EXCLUDE ( DOCTYPE,"ch" ) OR EXCLUDE ( DOCTYPE,"sh" ) OR EXCLUDE ( DOCTYPE,"bk" ) ) AND ( EXCLUDE ( EXACTKEYWORD,"Nonhuman" ) OR EXCLUDE ( EXACTKEYWORD,"Animal" ) OR EXCLUDE ( EXACTKEYWORD,"Mouse" ) OR EXCLUDE ( EXACTKEYWORD,"Animal Experiment" ) OR EXCLUDE ( EXACTKEYWORD,"Mice, Inbred BALB C" ) OR EXCLUDE ( EXACTKEYWORD,"Animal Tissue" ) OR EXCLUDE ( EXACTKEYWORD,"Bagg Albino Mouse" ) )

=625 (68 reviews)

**Web of Science 11/15/21**

#1

TI=(Vaccine* OR Vaccination* OR Pfizer OR Moderna OR immunization* OR variolation* OR "immunologic stimulation" OR Immunostimulation OR Gamaleya OR "Sputnik V" OR Sinovac OR "Corona Vac" OR AstraZeneca OR Janssen OR "AZD1222" OR "mRNA-1273" OR Janssen OR "Johnson & Johnson" OR "JNJ-78436735")

 #2

TS= (covid OR "Covid 19" OR "Corona virus" OR Coronavirus OR 2019nCoV OR SARSCoV2 OR 2019-nCoV)

#3

TI=(Effective* OR efficacy OR "antibody response" OR "antibody levels" OR IgG OR "neutralizing antibodies" OR success OR protect* OR prevent*)

#4

TI=(hesitance OR hesitancy OR hesitation OR attitude OR attitudes OR acceptance OR compliance OR accept OR acceptability OR perception OR perceptions OR influence OR influencing OR intent OR intention OR intentions OR perspective OR perspectives OR opinion OR opinions OR willingness OR receptivity OR barriers OR barrier OR motivation OR behavior* OR belief* OR questionnaire* OR survey* OR "case report" OR adverse OR "monoclonal antibodies" OR decision OR decisions OR pregnant OR pregnancy OR cost OR economic OR development OR supply OR distribution OR allocation OR prioritization OR mandate OR mandatory OR policy OR policies) OR TI=(nonhuman OR rat* OR mice OR mouse OR swine OR porcine OR "animal model" OR canine OR canines OR dog OR dogs OR veterinary OR rodent OR rodents OR porcine OR swine OR primate OR primates OR macaques OR ‘animal models’

(#1 AND #2 AND #3) NOT #4= 752 (79 reviews)

**Cochrane 11/15/21**


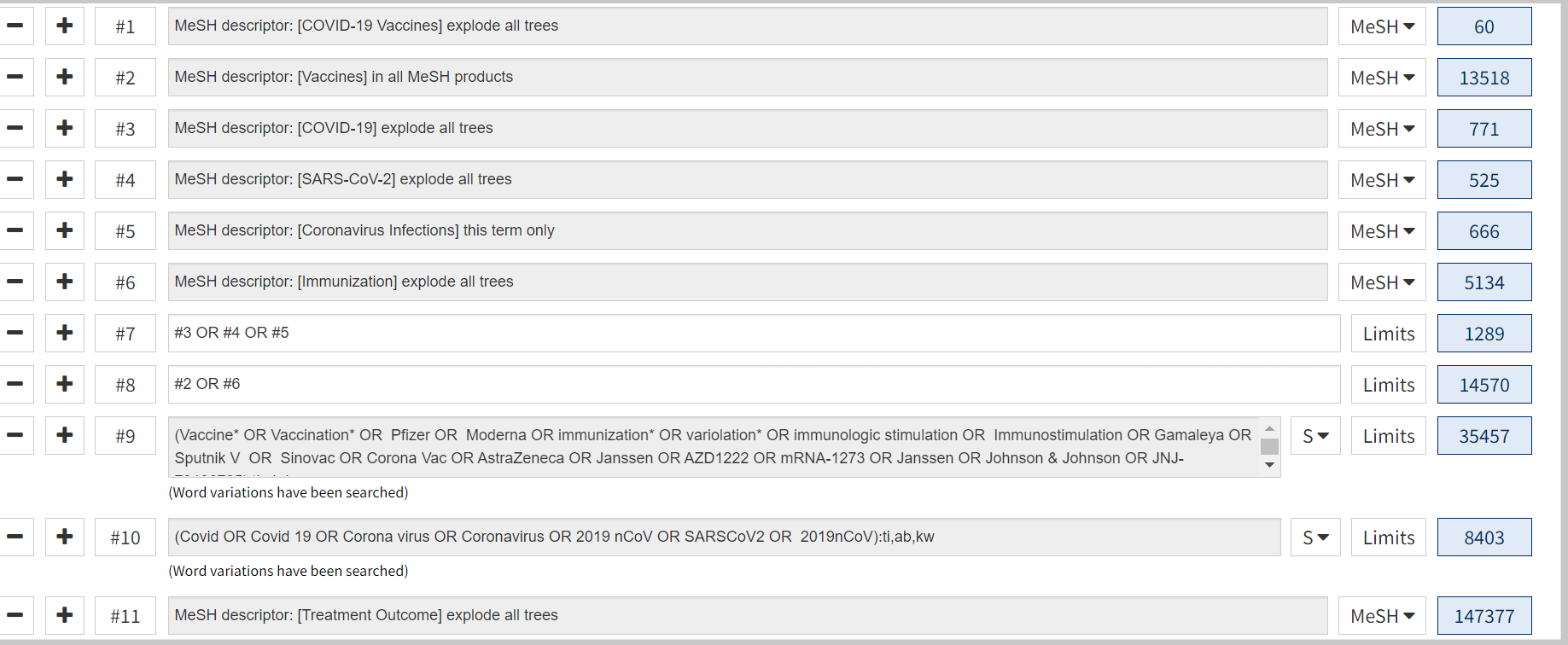


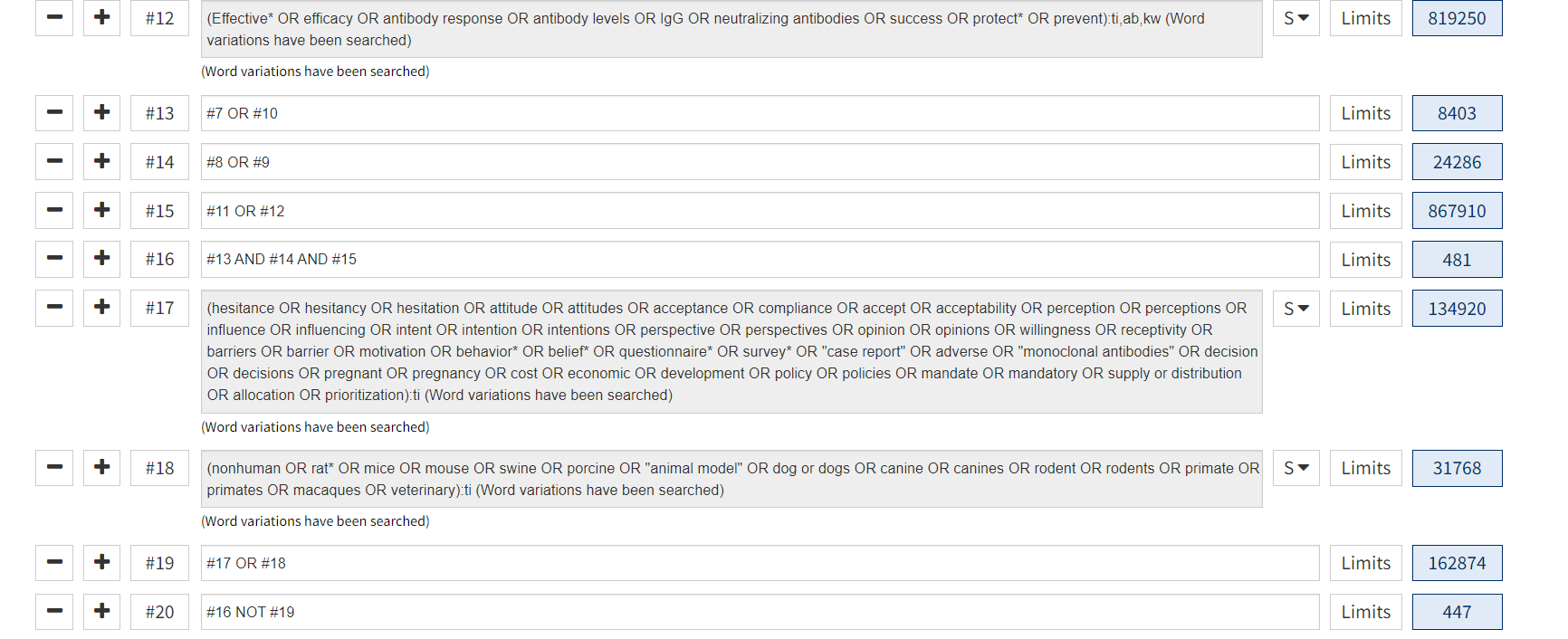


**447, 2 reviews**
